# A semi high-throughput method for screening small bispecific antibodies with high cytotoxicity

**DOI:** 10.1038/s41598-017-03101-4

**Published:** 2017-06-06

**Authors:** Aruto Sugiyama, Mitsuo Umetsu, Hikaru Nakazawa, Teppei Niide, Tomoko Onodera, Katsuhiro Hosokawa, Shuhei Hattori, Ryutaro Asano, Izumi Kumagai

**Affiliations:** Department of Biomolecular Engineering, Graduate School of Engineering, Tohoku University, 6-6-11 Aoba, Aramaki, Aoba-ku, Sendai, 980-8579 Japan

## Abstract

Small bispecific antibodies that induce T-cell–mediated cytotoxicity have the potential to damage late-stage tumor masses to a clinically relevant degree, but their cytotoxicity is critically dependent on their structural and functional properties. Here, we constructed an optimized procedure for identifying highly cytotoxic antibodies from a variety of the T-cell–recruiting antibodies engineered from a series of antibodies against cancer antigens of epidermal growth factor receptor family and T-cell receptors. By developing and applying a set of rapid operations for expression vector construction and protein preparation, we screened the cytotoxicity of 104 small antibodies with diabody format and identified some with 10^3^-times higher cytotoxicity than that of previously reported active diabody. The results demonstrate that cytotoxicity is enhanced by synergistic effects between the target, epitope, binding affinity, and the order of heavy-chain and light-chain variable domains. We demonstrate the importance of screening to determine the critical rules for highly cytotoxic antibodies.

## Introduction

Antibody molecules with high molecular recognition ability and cellular cytotoxicity have been extensively used as molecular-targeting agents. Their functions are independently localized in different fragments of the antibody: the fragment of variable region (Fv) has high specificity for the binding to target antigen or epitope, and the fragment of crystallizable region induces the activation of immune cells. This modular structure enables us to reconstruct antibodies with novel structures and functions that do not occur in nature.

Bispecific antibodies are non-natural antibodies reconstructed from two distinct monoclonal antibodies. The two different Fvs in a bispecific antibody simultaneously bind to two target antigens, and the formation of linkages between the two target antigens on cell surfaces can induce synergistic signals in the cells: for example, a bispecific antibody can induce blood clots by simultaneously binding to Factor IXa and Factor X^1^. For cancer therapy, cross-linking of immune cells with cancer cells induces the immune cells to damage the cancer cells. Especially, bispecific antibodies can target highly cytotoxic T cells, which are not activated by natural antibodies because T cells have no Fcγ receptors. Because of their abundance, proliferation capacity, and serial killing action, T cells can effectively attack tumors^2–4^; furthermore, previous reports have demonstrated that a bispecific T-cell–recruiting antibody can circumvent the mechanisms used by tumors to escape from immune effectors^5^.

First-generation bispecific antibodies were produced by means of hybrid hybridomas or chemical cross-linking; however, both these approaches generated populations of antibody molecules with heterogeneous structural properties^6–8^, which led to insufficient efficacy in the clinical setting. Advances in recombinant approaches have enabled the production of homogenous bispecific antibody molecules, of which several show efficacy in clinical trials. One of the advantages of the recombinant approach for bispecific antibody design is the downsizing of antibody because the bispecific function can be generated by using Fvs only. Although clearance of the small reconstructed antibodies from blood is faster than that of the natural antibodies^9^, the compact structure of the reconstructed antibodies contributes to low immunogenicity and high penetration into the tumor mass^10–12^. Several bispecific small antibodies with high T-cell–inducing cytotoxicity have been used in clinical trials^13–15^. In addition, these small antibodies have the potential to be produced by bacterial expression systems^16^, which would enable low-cost production of therapeutic antibodies.

These potential advantages of small T-cell–recruiting antibodies have driven researchers to generate a large number of these antibodies with different cancer targets and bispecific structure formats; the studies have shown that the cytotoxic activities of these antibodies depend on the antigen target and the antibody structure format^17^; for instance, changing the target can cause a ~10^3^-fold difference in cytotoxicity^18, 19^ and the cytotoxicity is strongly dependent on the bispecific structure (diabody, single-chain diabody, tandem single-chain Fv, etc) and arrangement of antibody domains^20, 21^. However, the relationships between these factors are complicated, and we have no optimized approach for choosing the appropriate Fvs and domain arrangements to construct bispecific antibodies with sufficiently high cytotoxicity to be clinically effective.

Here, we constructed a variety of bispecific T-cell–recruiting antibodies from a series of the Fvs against T-cell receptors (CD3 and CD28) and the epidermal growth factor receptor (EGFR) family (EGFR, HER2–4), and critical rules of high cytotoxic antibodies are elucidated in the screening process from the clump of bispecific antibodies. We focused on the traditional diabody, which has two single-chain Fv (scFv) fragments with swapped heavy-chain variable (VH) and light-chain variable (VL) domains dimerized to form bispecific antibodies. For each target epitope, we constructed diabodies with the VH and VL domains in different orders, because changing domain arrangement in a diabody can cause a more than 10^3^-fold cytotoxicity difference^21^. We developed a set of rapid operations for constructing the expression vectors and for expressing and purifying proteins, to make a variety of 100 diabodies with different hetero scFvs and domain arrangements. These prepared diabodies were then screened for high cytotoxicity in 3-(4,5-dimethylthiazole-2-yl)−5-(3-carboxymethoxyphenyl)−2-(4-sulfophenyl)−2H-tetrazolium inner salt (MTS) assays to ascertain the critical rules for design of antibodies with high cytotoxicity. The results showed the relationship between structural and functional properties and cytotoxicity of diabodies, in particular, the critical dependence of cytotoxicity on epitopes, binding affinity, and domain arrangement.

## Results

### Diabody library

To prepare the diabody-type bispecific antibody library, we used 4 anti-T-lymphocyte Fvs with affinity for CD3 or CD28, and 13 anti-cancer Fvs with affinity for members of the EGFR family (EGFR, HER2–4) (Table 1)^18, 22–39^. By changing the fusing orders of the VH and VL domains, two structurally different diabodies were constructed from each pair of Fvs: the HL-type diabody (VH domain of first Fv fused at the N-terminus of the VL domain of the second Fv via a peptide linker of GGGGS) and the LH-type diabody (VL domain of the first Fv fused at the N-terminus of VH domain of the second Fv via the peptide linker of GGGGS). Consequently, 52 diabodies were prepared in each HL-type and LH-type diabody group (Table 2), to give a total of 104 diabodies.

**Table 1:** Antibodies used to construct diabodies.

(A) Anti-CD3/CD28 antibodies				(B) Anti-EGFR family antibodies							
Name	Target	Epitope	Reference No.	Name	Target	Epitope	Reference No.	Name	Target	Epitope	Reference No.
L2K	CD3	ε chain	22, 23	7A7	EGFR	EGFR III	28	425	EGFR	EGFR III	34
OKT3	CD3	ε chain	24	175	EGFR	EGFR III	29	528	EGFR	EGFR III	18, 35
UCHT1	CD3	ε chain	25, 26	225	EGFR	EGFR III	30, 31	11F8	EGFR	EGFR III	36
9.3	CD28	N.D.^a^	27	806	EGFR	EGFR III	29	2C4	HER2	domain II	37, 53
				DL11	EGFR	EGFRIII	32	4D5	HER2	domain IV	38
					HER3	HERIII		A5	HER3	HER3 ECD	39
				h-R3	EGFR	EGFR vIII	33	B6	HER4	Unknown	39

^a^N.D.: Not determined.

**Table 2:** Combination of the Fv fragments used to construct diabodies.

		Target	Fv of anti-EGFR family antibody												
			7A7	175	225	806	DL11^a^	h-R3	425	528	11F8	2C4	4D5	A5	B6
			EGFR	EGFR	EGFR	EGFR	EGFR (HER3)	EGFR	EGFR	EGFR	EGFR	HER2	HER2	HER3	HER4
Fv of anti-CD3 or CD28 Antibody	L2K	CD3	1	2	3	4	5	6	7	8	9	10	11	12	13
	OKT3	CD3	14	15	16	17	18	19	20	21	22	23	24	25	26
	UCHT1	CD3	27	28	29	30	31	32	33	34	35	36	37	38	39
	9.3	CD28	40	41	42	43	44	45	46	47	48	49	50	51	52

Each diabody is labeled by the number listed in this table. ^a^DL11 is an antibody against EGFR and HER3.

For briefly constructing the expression vectors of diabodies, 4 VH genes containing were prepared from the vectors of anti-CD3 or -CD28 scFv, and they were simultaneously ligated into the 13 linearized vectors where VH genes were removed from anti-cancer scFvs with variable domains in the order VH-VL (Fig. S1A). The vectors containing hetero scFv genes with an anti-EGFR-family VH domain followed by an anti-CD3 or -CD28 VL domain were also produced in the same way, and all of the gene fragments encoding hetero scFv were ligated into the linear vector carrying the complementary hetero scFv to produce the expression vectors of HL-type diabodies. For LH-type diabodies, the expression vectors of anti-cancer VH domain were first produced and the anti-CD3 or –CD28 VL genes were simultaneously ligated into the linearized vectors of anti-cancer VH domain (Fig. S1B). A similar series of steps was used to produce the vectors containing hetero scFv genes with an anti-EGFR-family VL domain followed by an anti-CD3 or anti-CD28 VH domain, and all of the gene fragments encoding hetero scFvs were ligated to produce the vectors of LH-type diabodies.

### Screening library for active diabodies: direct MTS assay of culture solution

For convenient screening of these 104 diabodies to identify active candidates, we analyzed the cytotoxicity of the diabodies against TFK-1 (human bile duct carcinoma) cells in MTS assays by directly assaying the culture supernatant of the transformed *Escherichia* (*E*.) *coli* cells. In MTS assay of this study, growth inhibition of TFK-1 cells was calculated in the coexistence of lymphokine-activated killer cells with the T-cell phenotype (T-LAK cells). To estimate the influence of media components on the cancer cell damage in the MTS assay, we compared the cytotoxicity of *E. coli* culture supernatant without diabody to those with the HL-21 diabody, which has moderate cytotoxicity^18^ and can be expressed in culture supernatant (1 mg/media-L) (Fig. S2). When TFK-1 cells were mixed with undiluted diabody-free culture supernatant, the supernatants showed cytotoxicity; however, diabody-free culture supernatant that was diluted more than 10-fold showed no cytotoxicity. In contrast, 10- to 100-fold diluted culture supernatants from *E. coli* expressing the HL-21 diabody damaged most of the TFK-1 cells. These results indicate that dilution of culture supernatant at more than 10-fold can be used to avoid the influence of cytotoxic media components.

**Figure 1:**
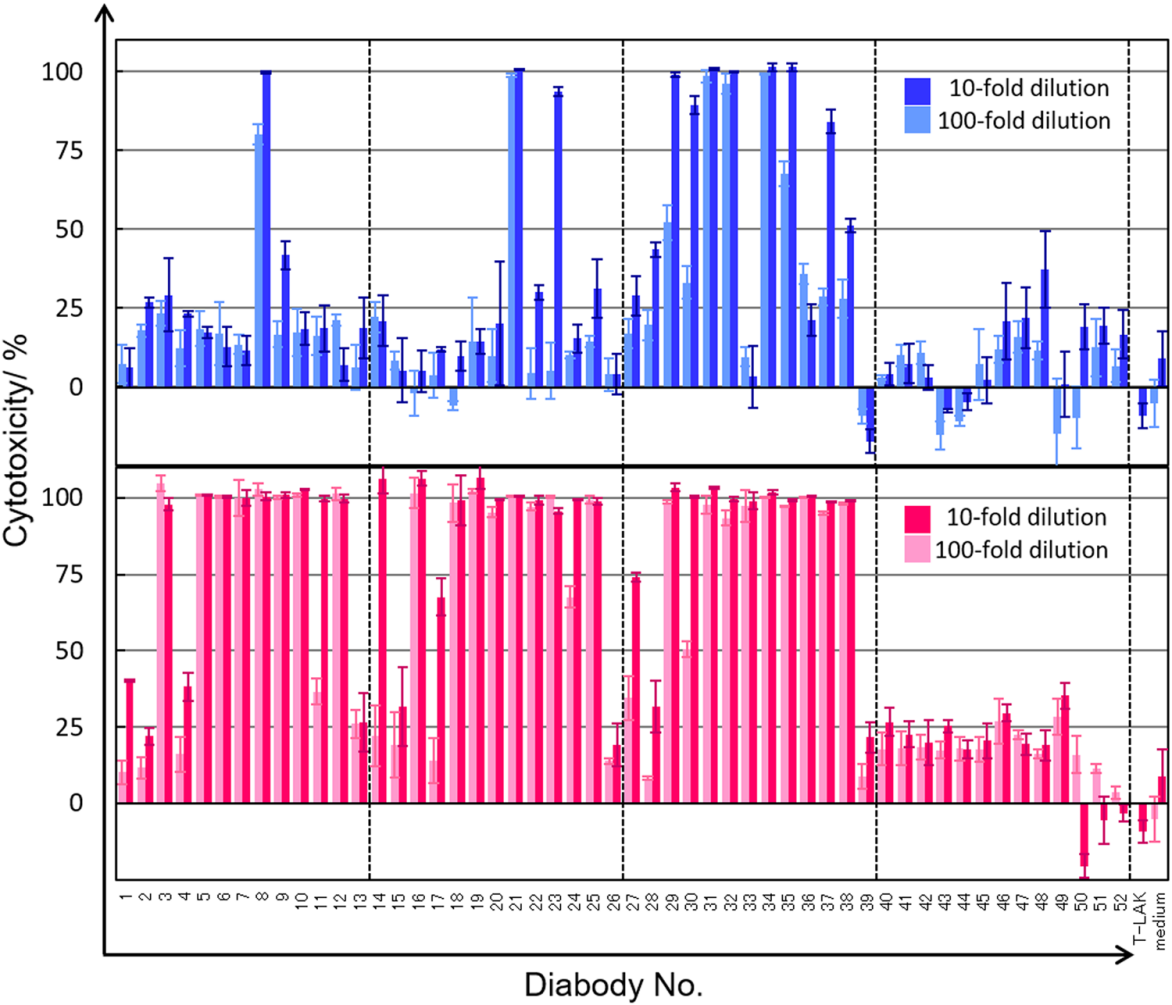
Cytotoxicity screening for culture supernatants of transformed *E*. *coli* against TFK-1 cells 10-fold or 100-fold diluted culture supernatants containing HL-type diabody (blue bar) or LH-type diabody (red bar) were applied to MTS assay. All the experiments were conducted three times, and data are presented as means ± S.D. In the horizontal axis, T-LAK indicates experiments without diabody but with T-LAK cells, and medium indicates experiments without diabody or T-LAK cells. Details of the diabodies nos 1–52 are presented in Table 2.

Figure 1 shows the results of the MTS assays of 10-fold and 100-fold diluted culture supernatants containing each of the 104 diabodies constructed here. Each of diabody-expressing *E. coli* was grown in a 500 mL-sized shake flask containing 250 mL medium, and the culture supernatants were used. Overall, a higher degree of cytotoxicity against TFK-1 cells was achieved with LH-type diabodies than with HL-type diabodies. Both HL-type and LH-type diabodies with anti-CD28 domains (i.e., diabodies nos 40–52, Fig. 1) caused only a low degree of cytotoxicity, whereas LH-type diabodies with domains from any of the three anti-CD3 Fvs (i.e, diabodies nos 1–39, Fig. 1) caused a substantial degree of cytotoxicity. However, HL diabodies containing the anti-CD3 domains from the UCHT1 Fv (i.e., diabodies nos 27–39, upper panel, Fig. 1) showed relatively high activity compared with those containing domains from other anti-CD3 Fvs.

### Quantitative screening for active diabodies: MTS assay of fraction from immobilized metal affinity chromatography

**Figure 2:**
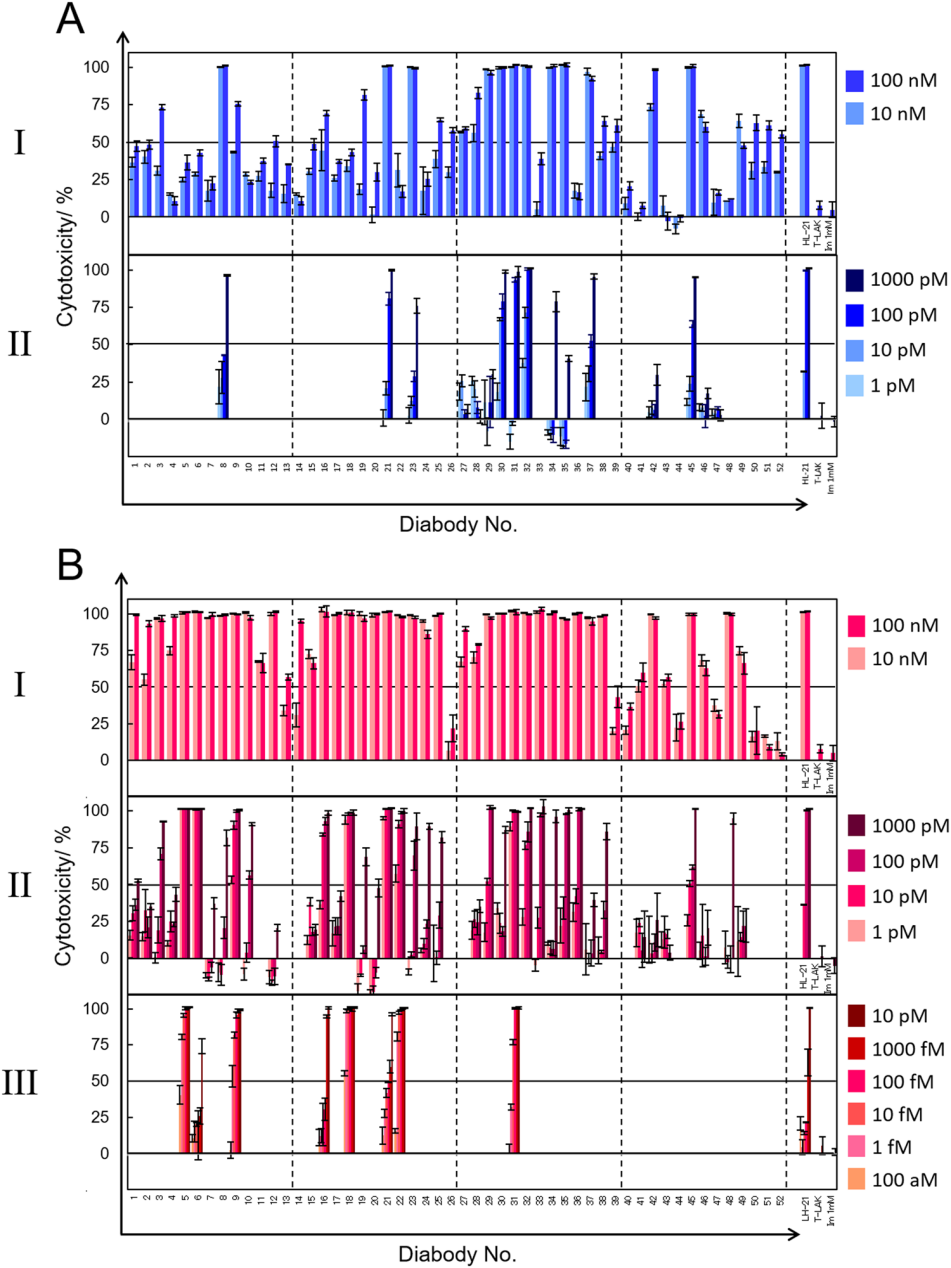
Cytotoxicity screening for IMAC-refined diabodies. (**A**) HL-type diabodies were added at concentrations of 10 nM or 100 nM (I), or 1 pM to 1 nM (II). (**B**) LH-type diabodies were added at concentrations of 10 nM or 100 nM (I), or 1 pM to 1 nM (II), or 0.1 fM to 10 pM (III). All the experiments were conducted three times, and data are presented as means ± S.D. In the horizontal axis, T-LAK indicates experiments without diabody but with T-LAK cells, and Im (1 mM) indicates experiments without diabody or T-LAK cells but with 1 mM imidazole. Details of the diabodies nos 1–52 are presented in Table 2.

The cytotoxicity estimated from direct MTS assay using culture supernatant is dependent not only on the cytotoxic activity of the diabody but also on the amount of diabody molecules expressed. Therefore, we then purified each diabody by means of immobilized metal affinity chromatography (IMAC) to estimate the diabody concentration, and we conducted a MTS assay of the eluted IMAC-refined diabody solution to quantitatively analyze the cytotoxic activity of each diabody (Fig. 2). To estimate the influence of imidazole, which is contained in the eluted solution, we analyzed the cytotoxicity of imidazole against TFK-1 cells in an MTS assay (Fig. S3); the results suggest that imidazole was cytotoxic at concentrations of more than 82 mM. Panel I in Fig. 2A shows the cytotoxicity of IMAC-refined HL-type diabodies (10 or 100 nM) against TFK-1cells. In this assay, each IMAC-refined diabody solution eluted by 300 mM imidazole was diluted to give a final diabody concentration of 10 or 100 nM. In these solutions, the final imidazole concentration was less than 82 mM (i.e., less than the concentration at which imidazole showed cytotoxicity in Fig. S3). In the MTS assays of IMAC-refined diabodies, damage to cancer cells by diabodies containing anti-CD3 domains was greater for LH-type than for HL-type diabodies, and diabodies containing anti-CD28 domains caused a low level of cancer cell damage. In these respects, the results for IMAC-purified diabodies were similar to those for diabodies in culture supernatants. However, some of the diabodies (HL/LH-42, HL/LH-45, and LH-48), which caused only minimal damage to cancer cells in the direct MTS assay of culture supernatant (Fig. 1), showed distinct cytotoxicity when purified and assayed at concentrations of 10 or 100 nM (Fig. 2), probably because the expression level of the diabodies in culture supernatant was low. This indicates that IMAC-refined diabodies can be used to reevaluate the cytotoxicity of diabodies with negatively estimates in the direct assay of culture supernatant.

For the 58 IMAC-refined diabodies with a cytotoxicity of more than 50% at 10 nM, we analyzed the activity at low concentration ranges: 1 pM to 1 nM (Fig. 2A,B panel II) and 0.1 fM to 10 pM (Fig. 2B, panel III). In the case of HL-type diabodies, cytotoxicity was not observed at less than 1 pM (data not shown). The HL-32 diabody, which showed the highest cytotoxicity of the HL-type diabodies, damaged 38% of cancer cells at 1 pM (Fig. 2A, panel II). In contrast, 8 LH-type diabodies (LH-5, -6, -9, -16, -18, -21, -22, and -31) showed more than 50% cytotoxicity at 1 pM (Fig. 2B, panel III). Although some diabodies were more cytotoxic in the HL-format, the LH-format was apt to be preferable for constructing highly cytotoxic diabodies: LH-format would be appropriate for TFK-1 cell.

**Figure 3:**
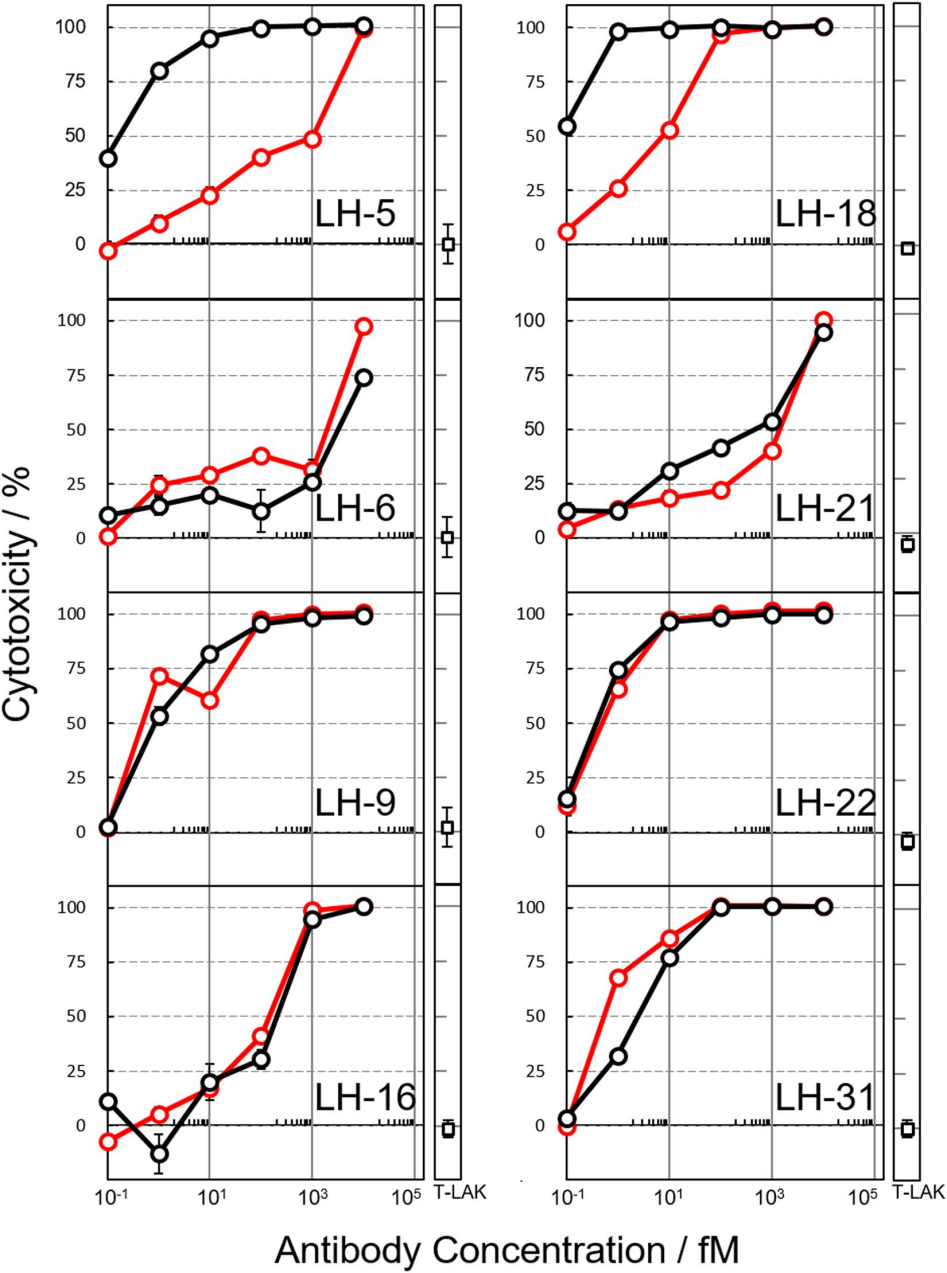
Comparison of cytotoxicity between IMAC-refined and SEC-purified diabodies. For each IMAC-refined diabody (black line) and SEC-purified diabody (red line) of LH-5, -6, -9, -16, -18, -21, -22, and -31, cytotoxicity against TFK-1 cells was measured. All the experiments were conducted three times. Data are represented as means ± S.E. In the horizontal axis, T-LAK indicates experiments without diabody but with T-LAK cells.

To more accurately estimate the dependence of cytotoxicity on diabody concentration, the 8 IMAC-refined diabodies with more than 50% cytotoxicity at 1 pM were further purified by means of size-exclusion chromatography (SEC) (Fig. S4), and the purified diabodies, which now contained no impurities, were examined by MTS assay (Fig. 3, red line). The differences in half maximum cytotoxicity concentration (IC_50_) between IMAC-refined and SEC-refined diabodies were less than 10-fold, except for diabodies LH-5 and -18. For highly cytotoxic diabodies, we conducted the reproducibility experiments of purification and cytotoxicity assay. As a result, all the SEC-refined diabodies had reproducible cytotoxicity, but cytotoxicity of the IMAC-refined diabodies LH-5 and -18 was not replicated: cytotoxicity of the IMAC-refined diabodies was similar to that of SEC-refined diabodies (Fig. S5). Therefore, the results of the MTS assay of IMAC-refined fractions for 104 diabodies possibly contained errors for several diabodies, probably attributed to impurities which was not occasionally removed by means of IMAC, but this method was effective for picking out highly cytotoxic diabodies.

### Analysis of half-maximal inhibitory concentrations

**Figure 4:**
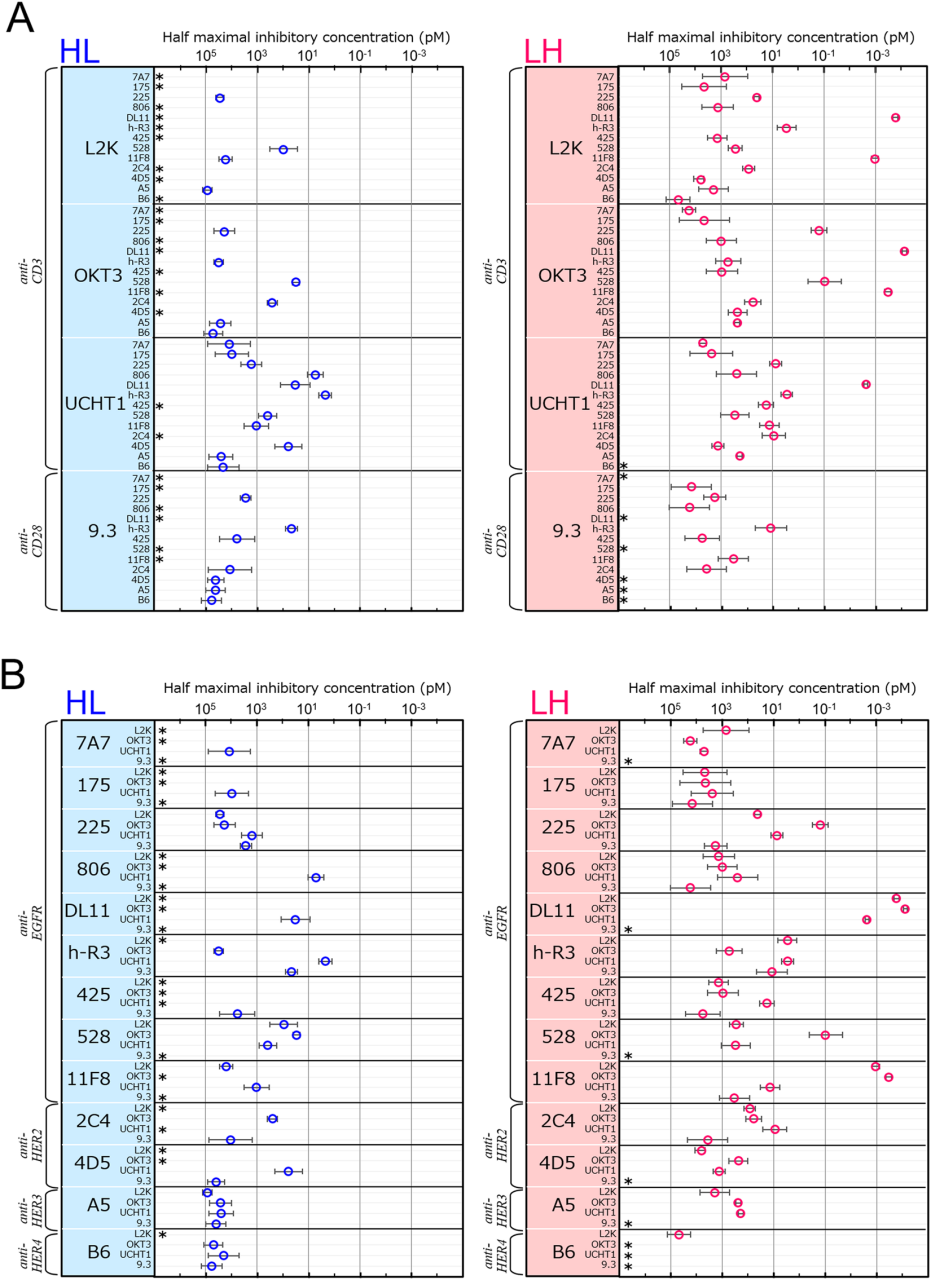
IC_50_ mapping of the entire diabody library to extract several rules for cytotoxicity IC_50_ values of the diabodies were grouped in terms of (**A**) the anti-T-lymphocyte Fv (i.e., anti-CD3 or -CD28 Fv), and (**B**) the anti-cancer Fv (i.e., anti-EGFR family Fv) used to construct the diabody. IC_50_ values were calculated from sigmoidal dose-response curve model, using PRISM software, and error bars represent 95% confidence intervals. Asterisks represent the diabodies with the cytotoxicity of less than 50% at 100 nM, because IC_50_ cannot be calculated.

Based on the results of the MTS assay of IMAC-refined fractions, we calculated IC_50_ values with 95% confidence intervals of all diabodies and compared the effect of domain order on cytotoxicity (Fig. 4). In the comparison between the HL-type and LH type diabodies constructed from the same Fvs, only 6 of 52 diabodies showed higher cytotoxicity in the HL-type than in the LH-type format; in contrast, 39 diabodies showed higher cytotoxicity in the LH-type format, indicating that the LH-type format generally showed superior cytotoxicity. In Fig. 4, the IC_50_ values are grouped in terms of the Fvs from which the diabodies were constructed (Fig. 4A; anti-T-lymphocyte, 4*B*; anti-cancer). In the IC_50_ maps grouped by anti-CD3 Fv (Fig. 4A, top three panels), many of the HL-type diabodies containing L2K- or OKT3-derived domains showed no cytotoxicity (i.e, IC_50_ ≧ 10^5^ pM (10 nM), Fig. 4A), whereas all of the corresponding LH-type diabodies had IC_50_ values less than 10^5^ pM. All but two of the diabodies containing UCHT1-derived domains showed higher cytotoxicity in the LH-type format than in the HL-type format; nevertheless, all the HL-type diabodies derived from UCHT1 except for two had IC_50_ values less than 10^5^ pM. The results suggest that the LH-type diabodies with anti-CD3 domains were apt to express high cytotoxicity, whereas the cytotoxicity of HL-type diabodies was dependent on the character of the anti-CD3 Fv used in their construction.

The cytotoxicity of diabodies derived from the anti-CD28 Fv (Fig. 4A, bottom panels) was lower than that of diabodies with anti-CD3 domains; however, both the HL-type and LH-type diabodies with anti-CD28 domains showed cytotoxicity against tumor cells and they had little difference in cytotoxicity between HL-type and LH-type. This suggests that the selection of the target on the T-lymphocyte (i.e., CD3 or CD28) influenced the cytotoxicity and the dependence of cytotoxicity on domain order.

In the IC_50_ maps grouped by anti-cancer fragment (i.e., anti-EGFR-family Fv) (Fig. 4B), most HL-type diabodies showed low cytotoxicity, and the cytotoxicity of the active HL-type ones was lower than that of the corresponding LH-type ones. Among the LH-type diabodies with anti-CD3 domains (L2K, OKT3, UCHT1), those constructed from DL11 or 11F8 Fv (against EGFR) had substantial cytotoxicity, with little dependence on the anti-CD3 Fv of the parental antibody; that is, except for the diabody with 11F8- and UCHT1-derived domains, the IC_50_ value was more than 10 pM. Therefore, even if the target on the cancer cell was the same, changing the Fv used to construct the diabody critically influenced the cytotoxicity.

In conclusion, among the diabodies constructed from the anti-cancer cell and anti-T-lymphocyte Fvs, the diabody group with the highest cytotoxicity against TFK-1 cancer cells follows the following rules: 1) the target on the T-lymphocyte is CD3, 2) the domain order is LH-type, 3) the target on the TFK-1 cancer cells is EGFR, 4) the anti-EGFR domains are derived from DL11 or 11F8 Fv.

## Discussion

### Convenient screening of diabody from domain library

In this study, we performed rapid screening of diabodies constructed from a combination of anti-T-lymphocyte and anti-cancer Fvs to identify diabodies with high cytotoxicity against cancer cells. To maximize the speed of this screening process, we developed a method for high-throughput preparation of recombinant proteins by refining the steps of expression vector construction, protein expression, and protein purification. For instance, to simultaneously construct a number of expression vectors, we applied the in-one-pot-at-a-time (iPaT) ligation approach^40^. Gene fragments encoding a VH and VL domain were prepared from scFv expression vectors, and a mixture of them were introduced into linearized expression vectors by iPaT ligation to simultaneously generate each diabody expression vector (Fig. S1). This method can be used to construct diabody expression vectors from scFv clones selected by means of molecular evolution, such as clones selected from scFv-displaying phages libraries.

In addition, the property that diabodies expressed in *E. coli* are secreted into culture enabled us to bypass protein purification process. 10- or 100-fold dilution of the culture supernatant led to negligible contamination by toxic culture components in the MTS assay. The cytotoxicity of diabodies in culture supernatant and IMAC-refined fraction were qualitatively comparable in the MTS assay, with some exceptions due to low concentration of diabodies in culture supernatant. Therefore, if the screening for active diabodies was enough by only direct MTS assay of culture supernatant, 96-deep well plate would be applied for our semi high-throughput method. The *E. coli* growth rate difference dependent upon the location of plate causes variation of the diabody concentration from well to another; as a result, some active diabodies might be missed in the screening using the deep well plates. By applying the ELISA method for estimating qualitative amount of diabody, the probability of missing is possibly decreased. The combination of the selection of active scFv clones from libraries and the rapid screening might open the way to high-throughput identification of diabodies with high cytotoxicity.

While the effectiveness of the MTS assay of culture supernatant and IMAC-refined fraction was shown, a series of the results possibly had several incorrect data; in this study, two among 8 IMAC-refined diabodies showed higher cytotoxicity than SEC-refined diabodies (Fig. 3). It is probably attributed to residual impurities which would not be occasionally removed by means of IMAC. Residual endotoxin can damage cancer cells, and synergy effect between the cytotoxicity of impurities and diabodies might be generated. Therefore, the reproducibility of cytotoxicity of selected diabody candidates should be confirmed by the assay of SEC-refined diabodies.

Here, we applied the diabody format as a small bispecific antibody model, because active diabodies can be expressed in *E. coli*. Whereas, tandem-scFv format, where scFv is fused at the C-terminus of another scFv via a peptide linker, have stable structure, and the tandem scFvs with high cytotoxicity, called BiTE, have been used in clinical trials. In addition to Blinatumomab, anti-CD3 × anti-CD19 BiTE approved by the US food and Drug Administration, several BiTEs have been used in clinical trials^13–15^. Active tandem scFvs are hardly expressed in *E. coli*, but we have been trying to develop an *E. coli* expression process for preparing active tandem scFv antibodies. Application of this convenient screening process from domain library for tandem scFv would be advantageous for generating highly cytotoxic antibodies with various structure format.

### Rules for domain order for design of diabodies with high cytotoxicity

Here, we used two different domain orders (HL and LH) when constructing the diabodies; our results for diabodies with anti-CD3 domains indicate the dependency of cytotoxicity on domain order (Fig. 4A). Crystal structures of the diabodies constructed from only one kind of Fv have been reported for an HL-type diabody from anti-phospholipase antibody L5MK16^41^, and an LH-type one from anti-carcinoembryonic antigen antibody T84.66^42^. The HL-type diabody has a symmetric structure with 2-fold axes in each Fv fragment, and the Fv center-to-center axes are parallel; in contrast, the LH-type diabody is asymmetric and is more flexible than the HL-type diabody. The difference in domain order (HL versus LH) may results in differences in both the cross-linked structure formed between the two target molecules and the flexibility of the diabody. Recently, we reported that an HL-type diabody constructed from humanized anti-EGFR 528 and anti-CD3 OKT3 antibody shows lower cytotoxicity than an LH-type diabody with the same target affinities^21^. The HL-type and LH-type antibodies bind to each target with the same affinity when only one target was present; however, soluble EGFR is not bound by the HL-type diabody when the diabody is bound to CD3-displaying T-lymphocytes. We consider that T-cell receptor complexed with CD3 sterically inhibits the binding of soluble EGFR onto the HL-type diabody. Here, most of the LH-type diabodies with L2K- or OKT3-derived anti-CD3 domains showed higher cytotoxicity than the corresponding HL-type diabodies, whereas the cytotoxicity of the diabodies with anti-CD28 domains was comparable between HL- and LH-type formats (Fig. 4A). Therefore, the domain order of a diabody should be designed in relation to the target on the T-lymphocytes.

The binding affinity for targets should also be considered when designing diabodies with high cytotoxicity. In the case of LH- and HL-type diabodies constructed from the Fvs of 11F8 (anti-EFGR) and A12 (anti-insulin-like growth factor receptor) antibodies, the affinity for EGFR is higher for the LH-type format than for the HL-type format, and only the LH-type diabody has affinity for insulin-like growth factor receptor^43^. Similarly, LH-type, but not HL-type, diabodies with domains against kinase insert domain receptor and Fms-like tyrosine kinase have affinity for the targets^44^. In contrast, an HL-type diabody constructed from a B-cell–targeting antibody and anti-lymphoma idiotype antibody has higher affinity than the corresponding LH-type diabody^45^, and for diabodies constructed from humanized anti-EGFR 528 and anti-CD3 OKT3 antibodies, the domain order makes no difference to the affinity to either target^21, 46^. Therefore, some HL-type diabodies have comparable or higher affinity for the targets when compared to LH-type one, but in most studies, the LH-type format can express higher target affinity than HL-type. Choice of the domain order that results in optimal target affinity is likely one of the critical factors for constructing diabodies with high cytotoxicity.

### Rules for the anti-T-lymphocyte Fv in the design of diabodies with high cytotoxicity

Here, the use of LH-type diabodies with anti-CD28, not anti-CD3, domains to target T-cell receptors resulted in low cytotoxicity (Fig. 4A). The anti-CD28 antibody, 9.3, whose Fv fragment was used to construct the anti-CD28 diabodies, is superagonistic because it can activate T-lymphocytes without activation of the T-cell receptor–CD3 complex^47^. The bispecific tandem scFv that comprises the 9.3 antibody is cytotoxic to malignant B cells^48^ and melanoma cells^27^. The 9.3 antibody recognizes the C′′D loop in CD28^49^; the binding enables IgG-type antibody to make a linkage between two CD28 molecules, and the linkage is considered to be the trigger for activating the T-lymphocytes^49^. The dimerized form of the tandem scFv is reported to have higher cytotoxicity than the monomeric tandem scFv^27, 48^. Although we cannot rule out the possibility that the structure format is critical (diabody or tandem scFv) for activating T-lymphocyte via CD28, the monovalent form against CD28 in diabodies is insufficient for activating T-lymphocyte.

### Rules for the anti-cancer Fv in the design of diabodies with high cytotoxicity

In the IC_50_ maps grouped by anti-cancer Fv (Fig. 4B), the LH-diabodies with anti-EGFR DL11 or 11F8 domains had very high cytotoxicity, with little dependency on the kind of anti-CD3 fragments used. Table S1 lists the reported binding affinities of the anti-EGFR parental antibodies used in this study^50^. In the group of LH-diabodies with anti-EGFR domains and IC_50_ values less than100 pM, those with high cytotoxicity were derived from the antibodies 225, DL11, h-R3, 425, 528, and 11F8 (group 1), which target folded EGFR (i.e., sEGFR [Table S1]), and their epitopes overlap with the area where EGF binds, and the others were derived from antibodies 7A7, 175, 806 (group 2), which target denatured EGFR fragments. Further, among the group 1 antibodies, DL11 and 11F8, which provided the domains for the diabodies with the highest cytotoxicity, showed the highest affinity for EGFR (*K*_d_ values of 1.9 nM and 3.3 nM, respectively; Table S1), and the antibodies h-R3, 425, and 528, which provided the domains for the diabodies with the next highest cytotoxicity, showed high affinity for EGFR (*K*_d_ values from 10 nM to 100 nM). This implies that the affinity strength for the epitope overlapping with the EGF-bound area on folded EGFR correlates with cytotoxicity. We note that the affinity for EGFR of antibody 225 is similar to those of the antibodies DL11 and 11F8, but the diabodies constructed from 225 Fv had comparable cytotoxicity to those constructed using Fvs from group 2 antibodies. This result might be due to structural instability of 225-Fv–derived diabodies, because the expression level of these diabodies was much less than that of the other diabodies (data not shown).

To analyze the applicability of the cytotoxic rules for other cell lines, several SEC-refined diabodies with different cytotoxicity against TFK-1 cells were used for the MTS assay against A431 (human epidermoid carcinoma) cells (Fig. S6). Consequently, the relative cytotoxicity of diabodies against A431 cells was comparable to that against TFK-1 cells: that is, 4 highly cytotoxic diabodies against TFK-1 cells (LH-5, LH-9, LH-18, and LH-22) showed higher cytotoxicity against A431 cells than 2 intermediate cytotoxic diabodies (LH-21 and LH-33), and HL-type diabody (HL-21) was lower cytotoxic than LH-diabodies. The cytotoxic rules may be applicable for other cancer cells expressing EGFR.

In conclusion, a domain library approach generated various bispecific diabodies with a wide-range of cytotoxicity, and convenient screening of the generated diabodies led to the identification of those with high cytotoxicity. The results of this screening process demonstrate that cytotoxicity changes drastically according to the Fv used and the domain order, and provide critical rules for the design of diabodies with high cytotoxicity: 1) the target on the T-lymphocyte is CD3, 2) the domain order is LH-type, 3) the target on the TFK-1 cancer cells is EGFR, 4) anti-EGFR antibody with high affinity for the epitope overlapping with the EGF-bound area on folded EGFR should be used. In general, parental antibodies binding the desired target are selected from large-scale libraries, but combinatorial optimization of the choice of Fv fragments and domain order to construct highly cytotoxic bispecific antibodies has not been attempted previously. Our results reveal that the construction of a diabody library from parental antibodies, used in combination with our novel screening method enables the rapid selection of Fvs suitable for constructing highly cytotoxic bispecific antibodies.

## Methods

### Construction of expression vectors for diabodies

Seventeen expression vectors for anti-EGFR family, anti-CD3, or anti-CD28 scFvs with variable domains in the order VH-VL, linked via a glycine rich peptide (3 × GGGGS), were constructed by ligating the gene fragments encoding each scFv into linear pRA vectors digested with *Nco*I and *Sac*II.

The steps used to produce expression vectors for HL-type diabodies with affinity for both an EGFR family member and either CD3 or CD28 (Fig. S1A) are described below. Four gene fragments encoding each anti-CD3 or -CD28 VH domain were prepared from the corresponding scFv gene fragments in the pRA vectors by digestion with *Nco*I and *Bsp*EI. DNA encoding VH domains and a part of linker sequence (2 × GGGGS) was removed from the 13 pRA vectors for anti-EGFR family members by digestion with *Nco*I and *Bsp*EI to produce linearized vectors. Finally, the four digested fragments containing anti-CD3 or -CD28 VH domains were simultaneously ligated into the 13 VH-minus and linearized pRA vectors to replace the original VH domain^40^. *E. coli* bacteria were transformed with the resultant vectors to clone and separate each vector containing a hetero scFv gene with an anti-CD3 or -CD28 VH domain followed by an anti-EGFR-family VL domain. A similar procedure was used to produce clones of vectors containing hetero scFv genes with an anti-EGFR-family VH domain followed by an anti-CD3 or -CD28 VL domain. Then, all of the fragments encoding hetero scFvs were prepared by polymerase chain reaction, and each amplified fragment was ligated into the linear (*Spe*I- and *Eco*RI-digested) pRA vector carrying the complementary hetero scFv.

For LH-type diabodies (Fig. S1B), 13 gene fragments encoding anti-EGFR family VH domains were prepared by *Bam*HI and *Pst*I digestion of the corresponding pRA vectors. The gene fragments were mixed and then simultaneously ligated into pRA5 vectors linearized with *Bam*HI and *Pst*I; the resultant vectors contained the linker (3 × GGGGS) and VH in this order. These vectors were then cloned and separated. Four gene fragments encoding anti-CD3 or -CD28 VL domains were prepared by *Eco*RV and *Eag*I digestion of the corresponding pRA vectors. These gene fragments were then mixed and simultaneously ligated into the linearized pRA5 vectors containing the linker and anti-EGFR family VH domain, with removal of part of the linker sequence (2 × GGGGS). *E. coli* bacteria were transformed with the resultant vectors to clone and separate each vector containing a hetero scFv gene with an anti-CD3 or -CD28 VL domain followed by an anti-EGFR-family VH domain. A similar series of steps was used to produce clones of vectors containing hetero scFv genes with an anti-EGFR-family VL domain followed by an anti-CD3 or anti-CD28 VH domain. Finally, all of the fragments encoding hetero scFvs were prepared by polymerase chain reaction, and each amplified fragment was ligated into the linearized (*Spe*I- and *Eco*RI-digested) pRA vector with the complementary hetero scFv.

### Preparation of diabodies for *in vitro* cytotoxicity screening

*E. coli* strain BL21 Star (DE3) (Life Technologies) cells transformed with each expression vector were grown to the early stationary phase at 28 °C in a 500 mL-sized shake flask containing 250 mL of 2 × YT broth supplemented with 100 μg/mL ampicillin. To induce the expression of diabody, 0.5 mM isopropyl-1-thio-L-D-galactopyranoside was added when the optical density of the culture medium reached 0.8, and then the cells were grown overnight at 20 °C. The harvested cells were centrifuged (12000 × g, 2 h), and the supernatant was used for the cytotoxicity assay.

For the i*n vitro* cytotoxicity assay of diabodies directly in the culture supernatants (i.e., without purification), the solutions were sterilized by filtration (MILLEX GV 0.22 μm, Merck), and then 10- or 100-fold diluted with RPMI solution. Aliquots (50 μL) of the diluted solutions were used in the assay. *E*. *coli* strain BL21 Star (DE3) was also transformed with the pRA vector without the diabody gene, and the culture supernatant was used to evaluate the cytotoxicity of the culture solution without diabodies.

Diabody molecules in the culture supernatants were purified by means of IMAC. Immobilized diabody molecules in the IMAC column were washed with phosphate buffered saline (PBS) solution containing 50 mM imidazole, and then eluted with 300 mM imidazole solution. The IMAC-refined diabodies were filtered, and their concentrations were estimated by the absorbance at 280 nm. Various concentrations of these IMAC-purified diabodies were then examined for cytotoxicity.

Some IMAC-refined diabodies were further fractionated by using a SEC Hiload Superdex 200 prep-grade column (26/60, GE Healthcare Bio-Science), and the resultant SEC-purified diabodies were quantified by measuring the absorbance at 280 nm.

### *In vitro* cytotoxicity assay

Lymphokine-activated killer cells with the T-cell phenotype (T-LAK cells) were induced as previously described^51^. In brief, peripheral blood mononuclear cells were cultured for 48 h at a density of 1 × 10^6^ cells/mL in medium supplemented with 100 IU/mL of recombinant human interleukin 2 (IL-2; Shionogi Pharmaceutical Co.) in a culture flask (A/S Nunc) that was precoated with anti-CD3 monoclonal antibody (10 mg/mL). We used the human bile duct carcinoma (TFK-1) cell line, which was established in our laboratory^52^, as the target cells in this study. TFK-1 cells were cultured with RPMI 1640 medium supplemented with 10% fetal bovine serum, 100 U/mL penicillin, and 100 mg/mL streptomycin. *In vitro* growth inhibition of TFK-1 cells was measured with an MTS assay kit (CellTiter 96 Aqueous Nonradioactive Cell Proliferation Assay; Promega). The target cells (10,000 cells in 100 μL of culture medium) were plated on 96-well, half-area (A/2), flat-bottomed plates (Costar). Cells were cultured overnight to allow adhesion to the well. After removal of the culture medium by aspiration, 100 μL of T-LAK cells (effector cells) plus various concentrations of recombinant antibodies were added to each well, giving a final effector-to-target cell ratio of 5:1. After culture of the cells for 24 h at 37 °C, each well was washed with PBS three times to remove effector cells and dead target cells, and 90.5 μL of culture medium plus 9 μL of MTS and 0.5 μL of phenazine methosulfate solution (Promega) was added to each well. The plates were incubated for 1 h at 37 °C and then read on a microplate reader at a wavelength of 490 nm. Growth inhibition of target cells was calculated as described previously^51^: percentage growth inhibition of target cells = [1 − (*A*_490_ of experiment − *A*_490_ of background)/(*A*_490_ of control − *A*_490_ of background)] × 100%, where *A* is absorbance. From the correlation between antibody concentration and percentage growth inhibition, IC_50_ values with 95% confidence intervals were estimated according to sigmoidal dose-response curve model, using the software of PRISM ver.5.0 (GraphPad Software, U.S.A.).

## Electronic supplementary material

SUPPLEMENTARY INFO
