# Supplementary material for: A semi high-throughput method for screening small bispecific antibodies with high cytotoxicity
Source: Sci Rep. 2017 Jun 6;7:2862. doi: 10.1038/s41598-017-03101-4 (PMC5460266; doi:10.1038/s41598-017-03101-4)
Supplement: Supplementary file 1 — SUPPLEMENTARY INFO [file 41598_2017_3101_MOESM1_ESM.pdf]

[Supplementary Information]

A semi high-throughput method for screening small bispecific antibodies with high cytotoxicity

Aruto Sugiyama, Mitsuo Umetsu,* Hikaru Nakazawa, Teppei Niide, Tomoko Onodera, Katsuhiro Hosokawa, Shuhei Hattori, Ryutaro Asano, and Izumi Kumagai*

Figure S1

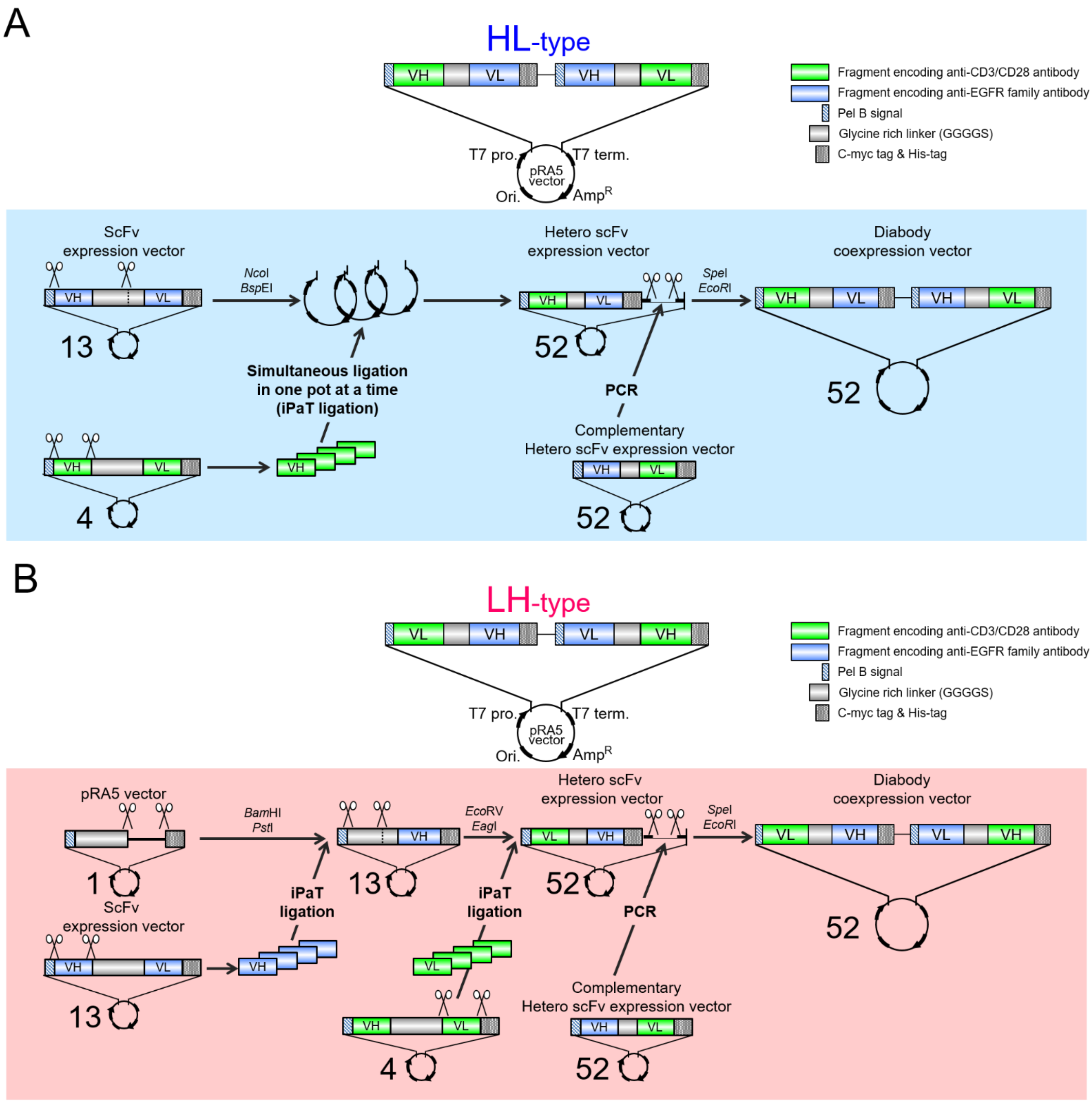

Figure S1 Construction of expression vectors for diabodies. (*A*) HL-type diabodies. (*B*) LH-type diabodies. T7 pro. indicates T7 promoter, T7 term. indicates T7 terminator, AmpR indicates ampicillin resistance gene, and Ori. indicates duplication origin.

Figure S2

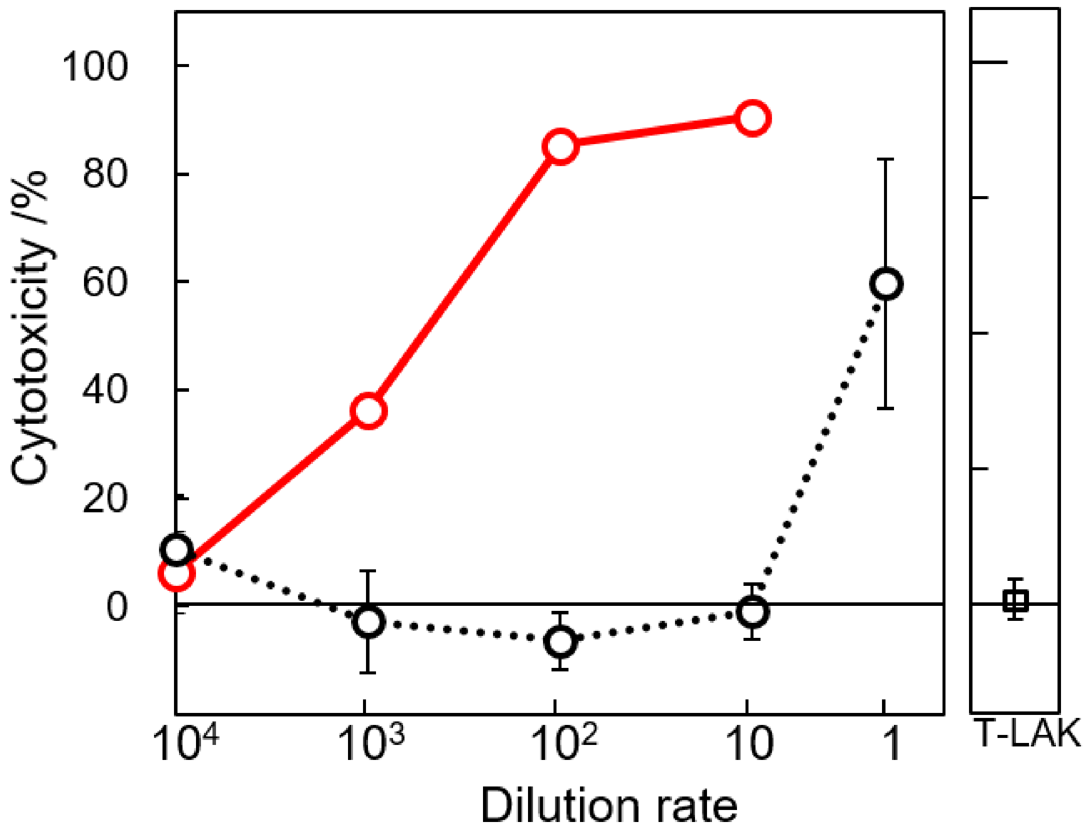

Fig. S2 Cytotoxicity of *E. coli* culture solution with diabody (red line) and without diabody (black dotted line), as determined by MTS. Data are presented as means ± 1 SD (n = 3) . T-LAK in the horizontal axis means that T-LAK was applied without diabody.

Figure S3

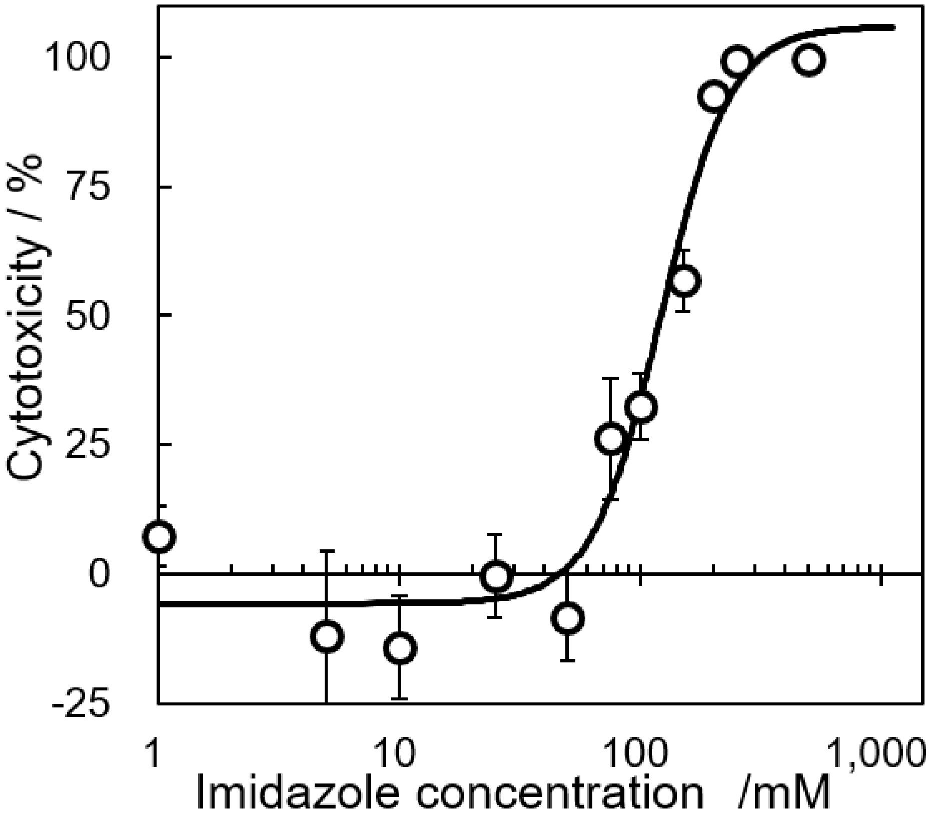

Fig. S3 Cytotoxicity of culture solution containing imidazole, as determined by MTS assay. The concentration of imidazole ranged from 1 mM to 500 mM. Data are presented as means ± 1 SD (n = 3)

Figure S4

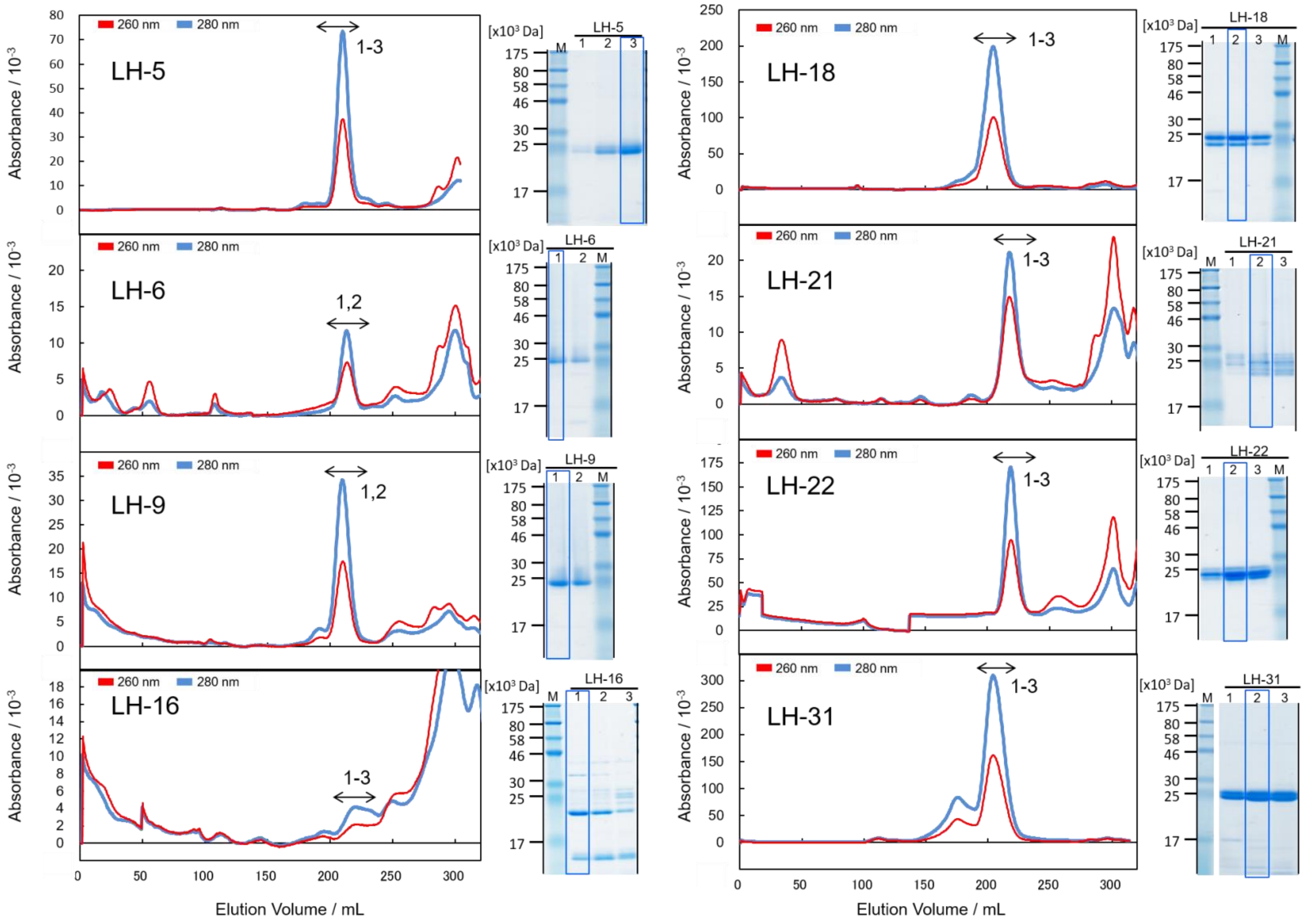

Fig. S4 Size-exclusion chromatograms for IMAC-refined diabodies of LH-5, -6, -9, -16, -18, -21,- 22, and -31, and SDS-PAGE results for fractionated diabodies. Red and blue lines in the chromatograms are the absorbance at 260 nm and 280 nm, respectively.

Figure S5

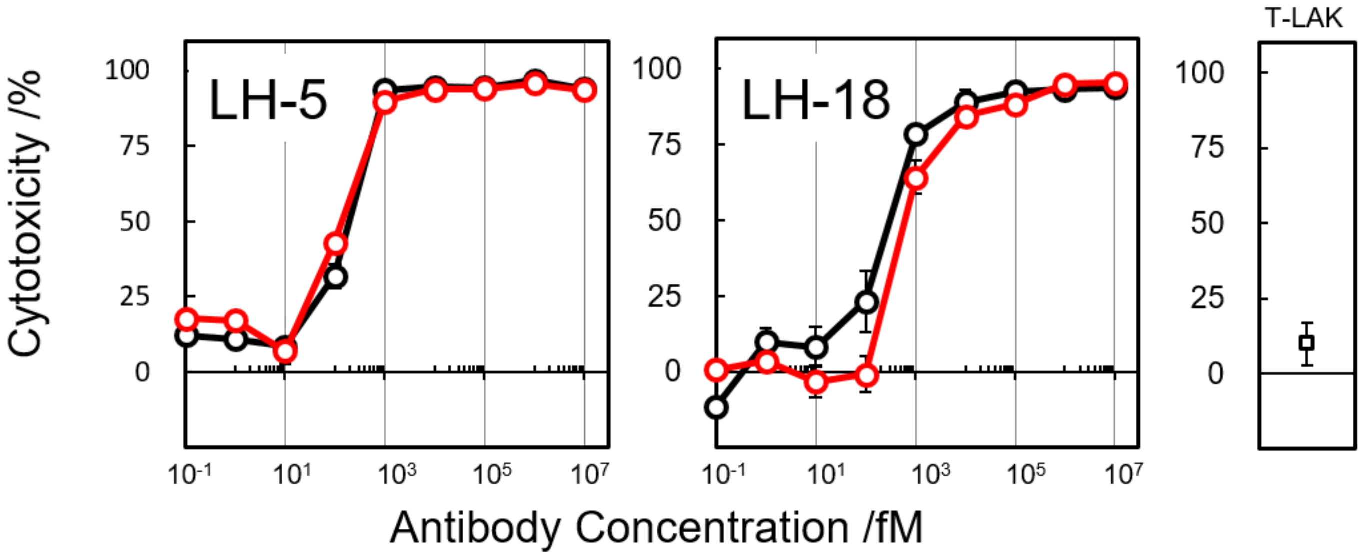

Fig. S5 Cytotoxicity assay against TFK-1 cells for IMAC-refined diabody (black line) and SEC-purified diabody (red line) of LH-5 and LH-18 to confirm reproducibility. All the experiments were conducted three times. Data are represented as means ± S.E. In the horizontal axis, T-LAK indicates experiments without diabody but with T-LAK cells.

Figure S6

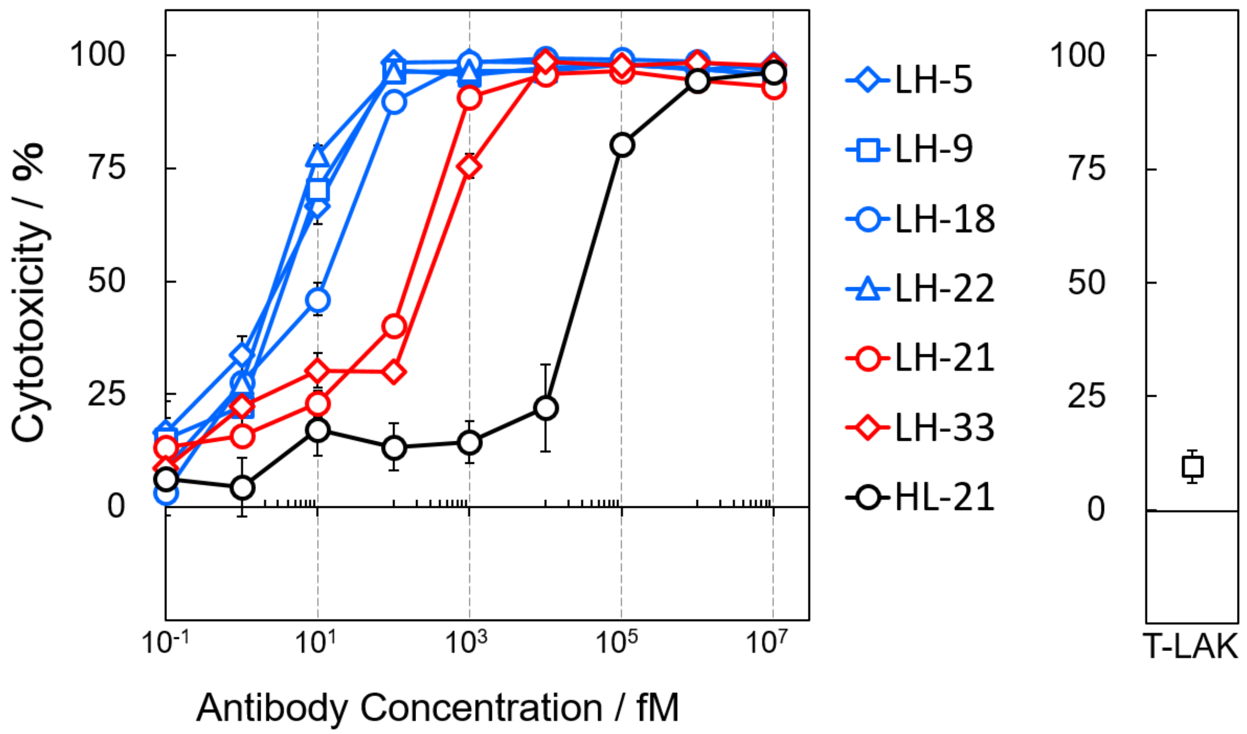

Fig. S6 Analysis of the applicability of the cytotoxic rules for other cell lines. Several SEC-refined diabodies with different cytotoxicity against TFK-1 cells were used for the MTS assay against A431 cells. 4 highly cytotoxic diabodies against TFK-1 cells (LH-5, LH-9, LH-18, and LH-22 are represented as blue line), 2 intermediate cytotoxic diabodies (LH-21 and LH-33 are represented as red line) and HL-type diabody, HL-21 (black line) were examined by MTS assay. All the experiments were conducted three times. Data are represented as means ± S.E. In the horizontal axis, T-LAK indicates experiments without diabody but with T-LAK cells.

Table S1.

Reported binding affinities of the anti-CD3/CD28 and anti-EGFR/HER2–4 antibodies

(A) Anti-CD3/CD28 antibodies

	Target	K_d (nM)	Format	Experimental target	Detection method	Reference number
**L2K**	CD3	100.0	scFv[b)]	n.d.	FCM[c)]	23
**OKT3**	CD3	200.0	diabody	CD3εγ	ITC[d)]	24
**UCHT1**	CD3	0.45	IgG	Jurkat cell	FCM	26
**9.3**	CD28	n.d. [a)]	-	-	-	

a) n.d.: Not determined, b)scFv: single chain Fv, c) FCM: flow cytometry, d)ITC: isothermal titration calorimetry

(B) Anti-EGFR/HER2–4 antibodies

	Target	K_d (nM)	Format	Experimental target	Detection method	Reference number
**7A7**	EGFR	n.d. [a)]	-	-	-	
**175**	EGFR	16	IgG	$EGFR_{(1-501)}$	SPR [e)]	29
		188.0		$EGFR_{(1-621)}$	SPR	29
**225**	EGFR	2.3	Fab [b)]	sEGFR [c)]	SPR	30
**806**	EGFR	34	IgG	$EGFR_{(1-501)}$	SPR	29
		389.0		$EGFR_{(1-621)}$	SPR	29
**DL11**	EGFR	1.9	Fab	sEGFR	SPR	32
	HER3	0.4	Fab	HER3	SPR	32
**h-R3**	EGFR	21.0	Fab	sEGFR	SPR	33
**425**	EGFR	113.0	Fab	sEGFR	SPR	34
**528**	EGFR	50.0	Fab	sEGFR	SPR	35
**11F8**	EGFR	3.3	Fab	sEGFR	SPR	36
**2C4**	HER2	8.5	Fab	HER2-ECD [d)]	SPR	53
**4D5**	HER2	0.1	IgG	HER2-ECD	ELISA	39
**A5**	HER3	160	tascFv	HER3-ECD	SPR	39
**B6**	HER4	n.d.	-	-	-	

a) n.d.: Not determined, b) Fab: Fragment, antigen binding, c) sEGFR: soluble EGFR, d) ECD: Extra cellular domain, e) SPR: surface plasmon resonance
